# Subcortical volumes in cerebral amyloid angiopathy compared with Alzheimer’s disease and controls

**DOI:** 10.3389/fnins.2023.1139196

**Published:** 2023-04-17

**Authors:** Chih-Hao Chen, Mary Klir Khnaijer, Andrew E. Beaudin, Cheryl R. McCreary, Myrlene Gee, Feryal Saad, Richard Frayne, Zahinoor Ismail, G. Bruce Pike, Richard Camicioli, Eric E. Smith

**Affiliations:** ^1^Department of Clinical Neurosciences, University of Calgary, Calgary, AB, Canada; ^2^Department of Neurology, National Taiwan University Hospital, Taipei, Taiwan; ^3^Hotchkiss Brain Institute, University of Calgary, Calgary, AB, Canada; ^4^Department of Radiology, University of Calgary, Calgary, AB, Canada; ^5^Division of Neurology, Department of Medicine and Neurosciences and Mental Health Institute, University of Alberta, Edmonton, AB, Canada; ^6^Department of Psychiatry, University of Calgary, Calgary, AB, Canada

**Keywords:** cerebral amyloid angiopathy (CAA), putamen, FreeSurfer, subcortical, volume measurements, atrophy

## Abstract

**Background:**

Previous reports have suggested that patients with cerebral amyloid angiopathy (CAA) may harbor smaller white matter, basal ganglia, and cerebellar volumes compared to age-matched healthy controls (HC) or patients with Alzheimer’s disease (AD). We investigated whether CAA is associated with subcortical atrophy.

**Methods:**

The study was based on the multi-site Functional Assessment of Vascular Reactivity cohort and included 78 probable CAA (diagnosed according to the Boston criteria v2.0), 33 AD, and 70 HC. Cerebral and cerebellar volumes were extracted from brain 3D T1-weighted MRI using FreeSurfer (v6.0). Subcortical volumes, including total white matter, thalamus, basal ganglia, and cerebellum were reported as proportion (%) of estimated total intracranial volume. White matter integrity was quantified by the peak width of skeletonized mean diffusivity.

**Results:**

Participants in the CAA group were older (74.0 ± 7.0, female 44%) than the AD (69.7 ± 7.5, female 42%) and HC (68.8 ± 7.8, female 69%) groups. CAA participants had the highest white matter hyperintensity volume and worse white matter integrity of the three groups. After adjusting for age, sex, and study site, CAA participants had smaller putamen volumes (mean differences, −0.024% of intracranial volume; 95% confidence intervals, −0.041% to −0.006%; *p* = 0.005) than the HCs but not AD participants (−0.003%; −0.024 to 0.018%; *p* = 0.94). Other subcortical volumes including subcortical white matter, thalamus, caudate, globus pallidus, cerebellar cortex or cerebellar white matter were comparable between all three groups.

**Conclusion:**

In contrast to prior studies, we did not find substantial atrophy of subcortical volumes in CAA compared to AD or HCs, except for the putamen. Differences between studies may reflect heterogeneity in CAA presenting syndromes or severity.

## Introduction

Cerebral amyloid angiopathy (CAA) is an age-related small vessel disease (SVD) primarily affecting the elderly population (Banerjee et al., 2017). Pathologically, progressive deposition of β-amyloid in the small- to medium-sized cortical and leptomeningeal vessels result in weakened cerebrovascular integrity which can cause cerebral microbleeds or overt intracerebral hemorrhage (ICH). Besides haemorrhagic brain insults, CAA also manifests with non-haemorrhagic injury such as chronic ischaemia, cerebral microinfarct, white matter damage, and structural disconnection (Smith, 2018; Greenberg et al., 2020). Collectively, CAA can lead to neurodegeneration and brain atrophy. A previous study has demonstrated cortical thinning in CAA patients compared to age-matched healthy controls (Fotiadis et al., 2016). Subsequent studies have reported subcortical white matter (Fotiadis et al., 2020), basal ganglia (Fotiadis et al., 2021), and cerebellar atrophy (Horn et al., 2022) in CAA participants compared to age-matched healthy controls and even patients with Alzheimer’s disease (AD). These findings suggested that CAA-related neurodegeneration may not only result in cortical atrophy, but also have independent effects on these subcortical structures.

Caveats regarding these prior studies include that all CAA participants were from the same single-hospital cohort, while the age-matched controls and AD participants were sampled from an external database, the Alzheimer’s Disease Neuroimaging Initiative (ADNI). Therefore, the validity of these observations requires external validation. Our study aims to compare the volumes of subcortical structures between CAA, AD, and controls from an ongoing cohort. We hypothesized that patients with CAA would have lower volumes in deep nuclei such as thalamus or basal ganglia because of subcortical ischemia and disconnection from the cortex.

## Methods

### Study population

The study was based on the ongoing, multi-site Functional Assessment of Vascular Reactivity (FAVR) study which consecutively recruited CAA, mild AD, and healthy control (HC) participants from Calgary and Edmonton, Canada (Peca et al., 2013; McCreary et al., 2020; Subotic et al., 2021; Beaudin et al., 2022). All participants were ≥ 55 years of age, did not have significant neurological or psychiatric disorders, or contraindications for MRI at 3 T. Participants with CAA and AD were mainly recruited from stroke prevention and cognitive clinics, while HCs were recruited from the community through poster advertisements or from spouses of patients. Participants provided informed consent prior to participation. The study protocol was approved by the University of Calgary and University of Alberta research ethics board.

Diagnosis of CAA was made according to the Boston criteria v2.0 (Charidimou et al., 2022). Clinically, CAA participants presented with ICH, transient focal neurological events, or mild cognitive impairment. Participants with CAA and recent ICH were assessed >90 days after symptomatic ICH to avoid confounding effects of acute hemorrhage. Participants with CAA-related inflammation were assessed during remission when no radiological evidence of cerebral edema was seen. In addition, potential CAA participants were excluded if they had dementia or lived in a long-term care facility. Participants with AD were diagnosed based on National Institute on Aging–Alzheimer’s Association criteria for mild dementia due to AD (McKhann et al., 2011). Healthy controls were screened by medical history and neuropsychological testing to exclude the presence of stroke, cognitive impairment or psychiatric illness. Of note, participants with AD or HCs were excluded if their MRI features were indicative of probable CAA according to the Boston criteria v2.0.

All participants completed a 1.25-h neuropsychological test battery, administered by qualified personnel. All test results were transformed into z-scores based on published norms of the healthy controls. Then the z scores were grouped into domains of memory, executive function, and processing speed. In the initial iteration of the study (*n* = 79), memory domain was derived from the Rey-Osterreith Complex Figure test (ROCFT) and the list A long free recall on the California Verbal Learning Test (CVLT-II; Shin et al., 2006; Woods et al., 2006); the executive function was based on the average of the Trail Making part B and the Control Oral Word Association Test-FAS (COWAT-FAS; Reitan, 1958; Lezak et al., 2004); the processing speed was based on the average of Trail Making part A and the Wechsler Adult Intelligence Scale (WAIS)-IV Digit Symbol-Coding (Reitan, 1958; Joy et al., 2004; Wechsler, 2008). In the second iteration of the study (*n* = 102), the ROCFT was replaced by the recall on trial 4 of the Brief Visuospatial Memory Test-Revised (BVMT-R), the CVLT-II was replaced by recall of the A7 word list of the Rey Auditory Verbal Learning Test (RAVLT), and the COWAT-FAS was replaced by the Letter Fluency raw score of the Delis-Kaplan Executive Function System (Homack et al., 2005; Schoenberg et al., 2006; Tam and Schmitter-Edgecombe, 2013).

### MRI acquisition

All participants underwent MRI on a 3 T scanner across two sites in Alberta, Canada in either Calgary [Signa VH/*I* with a 12-channel head coil or Discovery MR750 (GE Healthcare, Waukesha, WI) with a 32 channel Nova head coil (Nova Medical, Wilmington, MA)] or Edmonton (Siemens Prisma, Erlangen, Germany with a 20-channel head coil). The AC-PC line was used for head position alignment. Imaging sequences included inversion prepared 3D T1-weighted, T2-weighted fluid attenuated inversion recovery (FLAIR), susceptibility-weighted imaging (SWI), and diffusion weighted imaging. Imaging acquisition parameters are summarized in Supplementary Table 1.

### MRI visual rating

Visual rating of the cerebral SVD markers was performed by an experienced neuroradiologist blinded to the participant group. The presence of periventricular or subcortical white matter hyperintensity (WMH) were evaluated on FLAIR images and graded by the Fazekas scale for periventricular and subcortical regions (score 0–3, with higher score indicated more severe WMH; Fazekas et al., 1987). The severity of enlarged perivascular space (EPVS) was assessed on T2 images and graded by the Wardlaw scale (score 1–4, with higher score indicated more visible EPVS; Wardlaw et al., 2020). The presence, location and number of cerebral microbleeds (CMBs), as well as cortical superficial siderosis (cSS) were assessed on SWI. Finally, a total CAA-related SVD score was calculated based on the following: moderate to severe WMH burden (Fazekas ≥2 = 1 point), lobar CMBs (2–4 = 1 point; ≥5 = 2 points), cSS (focal = 1 point; disseminated = 2 points), and moderate to severe EPVS at centrum semiovale (>20 = 1 point); the total score ranged from 0 to 6 (Charidimou et al., 2016).

### MRI processing

Brain and intracranial volumes as well as cortical thickness were obtained by processing 3D T1 images with FreeSurfer (v6.0).^1^ FreeSurfer is a fully automated software used extensively in the neuroimaging field to measure structural volumetric properties of the brain (Fischl et al., 2004). In the present study, the regions of interest (ROI) included basal ganglia (putamen, caudate, globus pallidus), thalamus, cerebellar cortex, cerebellar white matter, cerebral cortex, and cerebral white matter. The subcortical structures and cerebellum were labeled and measured *via* the volume-based pipeline in FreeSurfer, and results were extracted from the output “*aseg.stats*.” We averaged left and right ROI volumes. For participants with an ICH, we used the results from the unaffected hemisphere only. The cerebral cortical volume was defined as the total volume inside the pial surface minus the volume inside the gray-white matter junction. The cerebral white matter volume was defined as the total volume inside the gray-white matter junction minus the volume of non-white matter structures such as the ventricles and subcortical gray matter. Estimated total intracranial volume (eTIV) and average cortical thickness were also derived from FreeSurfer. All FreeSurfer output were visually inspected for quality, and manual interventions (e.g., control points) were done when necessary. To control for the variations in head size, all volumetric data were expressed as a percentage of eTIV (% of eTIV).

The WMH volumes were measured on FLAIR images using a semi-automated, seed-based 3D region growing method (Cerebra Lesion Extraction Tool v1.0, Calgary Image Processing and Analysis Centre, Calgary, Canada; Kosior et al., 2011). The final segmented lesions were visually screened for accuracy. The WMH volumes were also expressed as % of eTIV. White matter integrity was quantified by the peak width of skeletonized mean diffusivity (PSMD) measured in units of × 10^−4^ mm^2^/s, analyzed by using the PSMD marker script^2^ and FSL version 6.0^3^ on the MRI diffusion images (Baykara et al., 2016). Higher values of PSMD indicate more severe cerebral white matter microstructural disruption. De-identified participant data and statistical code will be made available to other researchers upon reasonable request to the corresponding author.

### Statistical analysis

For participant characteristics, chi-square or Fisher’s exact tests were used for categorical variables, and analysis of variance (ANOVA) was used for continuous variables with Tukey’s correction for *post-hoc* comparisons. Because there existed significant differences of the age and sex between the three groups, and participants were recruited from two study sites, all FreeSurfer, WMH volumes and PSMD were analyzed using generalized linear regression models (PROC GLM in SAS) and expressed as age-, sex- and site-adjusted least square means and 95% confidence intervals. In the models, the dependent variable was subcortical volume, the independent variable was group, and the covariates were age, sex and study site. Least square means were compared between the three groups using an analysis of covariance (ANCOVA) including Tukey’s correction for multiple *post-hoc* group comparisons. The least square mean differences between groups were derived from the ANCOVA. Furthermore, we analyzed the association between subcortical volumes and cognitive domain z score. The multivariable linear regression model was applied, with cognitive domain z score as dependent variable, subcortical volume (expressed as 0.1 eTIV) as independent variables, and adjusted for age, female sex, diagnosis group, year of education, and study site. A term for the interaction between subcortical volume of interest and diagnosis group was also included in the models, but then these terms were removed because all of the interactions were not significant, indicating that the association of the subcortical volume with cognition did not vary by group. Because the volumes of right and left hemisphere subcortical structures were slightly, but significantly, different (data not shown) we performed an additional sensitivity analysis to test differences by hemisphere. The statistical significance level was *p* < 0.05 for all analyses. All analyses were performed using SAS, Version 9.4 (SAS Institute Inc., Cary, NC, United States).

## Results

**Figure 1:**
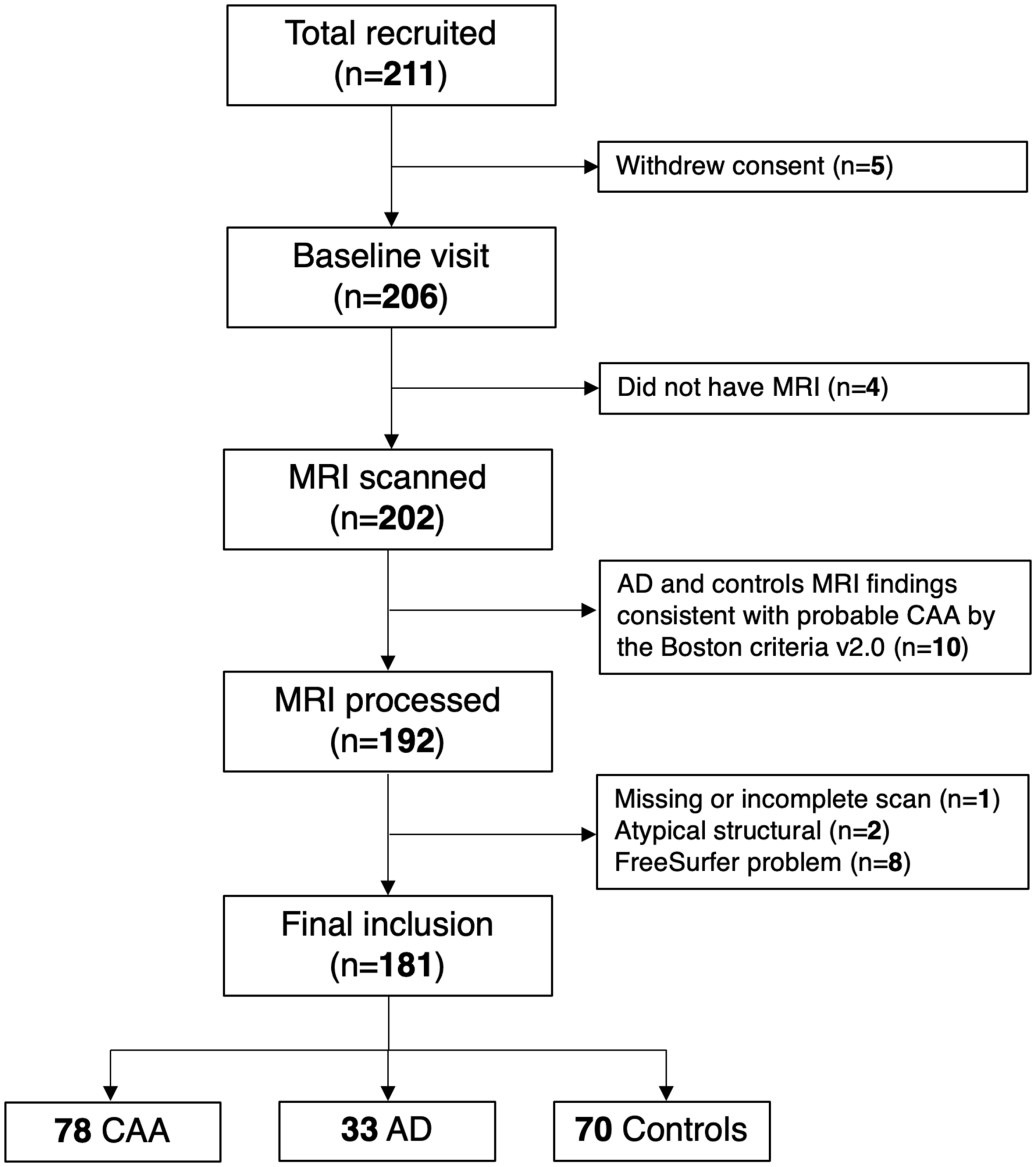
Study flowchart.

The study recruited 211 participants, with 202 undergoing MRI (5 withdrew consent; 4 did not undergo MRI). Of these, 10 AD and HCs were excluded because their MRI was consistent with probable CAA by the Boston criteria v2.0 after review by the neuroradiologist. The remaining 192 MRI scans were processed, of which 11 were excluded due to missing or incomplete scans, atypical structural abnormalities, or software problems. Thus, the final sample included 181 scans and consisted of 78 CAA, 33 AD, and 70 HCs (Figure 1). In the 78 CAA patients, 30 were recruited because of ICH, 19 because of mild cognitive impairment, and 29 because of transient focal neurological episodes.

CAA participants were older than AD and HCs (74.0 ± 7.0 vs. 69.7 ± 7.5 vs. 68.8 ± 7.8 years for CAA, AD, and HC, respectively; overall *p* < 0.001) and had fewer years of education (13.9 ± 2.9 vs. 15.4 ± 3.5 vs. 15.2 ± 3.1 years; *p* = 0.02), while HCs included more females (43.6% vs. 42.4% vs. 68.6%; overall *p* = 0.004). Regarding vascular risk factors, CAA participants had a higher proportion of hypertension (64.1% vs. 33.3% vs. 21.4%; overall *p* < 0.001; Table 1).

**Table 1:** Characteristics of included participants.

	CAA (*n* = 78)	AD (*n* = 33)	Controls (*n* = 70)	*p*-value
Age, years	74.0 ± 7.0	69.7 ± 7.5	68.8 ± 7.8	<0.001 ^a,b^
Female sex	34 (43.6%)	14 (42.4%)	48 (68.6%)	0.004
Education, years	13.9 ± 2.9	15.4 ± 3.5	15.2 ± 3.1 ^b^	0.02 ^b^
Hypertension	50 (64.1%)	11 (33.3%)	15 (21.4%)	<0.001
Diabetes mellitus	11 (14.1%)	3 (9.1%)	5 (7.1%)	0.370
Hyperlipidemia	35 (44.9%)	7 (21.2%)	28 (40.0%)	0.062
Ever smoker	43 (55.1%)	18 (54.6%)	30 (42.9%)	0.284
Site				0.051
Calgary	72 (92.3%)	29 (87.9%)	55 (78.6%)	
Edmonton	6 (7.7%)	4 (12.1%)	15 (21.4%)	
Cognitive z score				
Memory	−1.13 ± 1.10	−2.24 ± 0.80	0.52 ± 0.89	<0.001 ^a,b,c^
Executive function	−1.19 ± 1.13	−1.96 ± 0.96	0.28 ± 0.89	<0.001 ^b,c^
Processing speed	−0.88 ± 1.04	−1.48 ± 1.15	0.56 ± 0.83	<0.001 ^a,b,c^

All continuous data were presented in mean ± standard deviation and categorical data in number (%).

*P*-value provided for ANOVA; *Post-hoc p* < 0.05 in ^a^ CAA vs AD, ^b^ CAA vs controls, and ^c^ AD vs controls.

AD, Alzheimer’s disease; CAA, cerebral amyloid angiopathy.

### MRI visual rating

Participants with CAA had significantly higher Fazekas scale WMH scores, higher EPVS grade in the centrum semiovale, and more CMBs than AD and HCs (Table 2). Of note, more than half (59.0%) of CAA participants had visible cSS while only 1 AD and no HCs had cSS. Overall, CAA participants had significantly higher CAA-SVD score than AD and HCs (3.4 ± 1.3 vs. 0.4 ± 0.7 vs. 0.2 ± 0.1; *p* < 0.001).

**Table 2:** Summary of MRI cerebral small vessel disease markers.

Groups	CAA (*n* = 78)	AD (*n* = 33)	Controls (*n* = 70)	*p*-value
WMH Fazekas Scale				
Periventricular (0–3)	2.1 ± 0.8	1.2 ± 0.5	0.9 ± 0.7	<0.001 ^a,b^
Subcortical (0–3)	2.1 ± 0.8	1.2 ± 0.6	1.1 ± 0.5	<0.001 ^a,b^
EPVS-CSO severity (0–4)	2.0 ± 1.0	1.3 ± 0.6	1.5 ± 0.7	<0.001 ^a,b^
CMB (%)	76 (97.4%)	3 (9.1%)	7 (10.0%)	<0.001
CMB number	10 (3–48)	0 (0–0)	0 (0–0)	<0.001 ^a,b^
cSS (%)	46 (59.0%)	1 (3.1%)	0 (0.0%)	<0.001
Disseminated cSS (%)	19 (24.7%)	0 (0%)	0 (0%)	<0.001
Focal cSS (%)	26 (33.8%)	1 (3.1%)	0 (0%)	<0.001
CAA-SVD score (0–6)	3.4 ± 1.3	0.4 ± 0.7	0.2 ± 0.1	<0.001 ^a,b^

All continuous data were presented in mean ± standard deviation except for CMB number which is median (Interquartile range); all categorical data in number (%).

*Post-hoc p* < 0.05 in ^a^ CAA vs AD and ^b^CAA vs controls.

AD, Alzheimer’s disease; CAA, cerebral amyloid angiopathy; CMB, cerebral microbleeds; CSO, centrum semiovale; cSS, cortical superficial siderosis; EPVS, enlarged perivascular space; SVD, small vessel disease; WMH, white matter hyperintensity.

### Quantitative MRI analysis

Quantitative MRI markers are shown in Table 3. Overall, WMH volume was 1.84% of eTIV (95% CI, 1.54–2.16) for CAA, 0.70% (0.30–1.09) for AD, and 0.48% (0.20–0.76) for HCs; *post-hoc p* < 0.001 for CAA vs. AD and CAA vs. HCs. Meanwhile, PSMD was 4.02 × 10^−4^ mm^2^s^−1^ (3.74–4.30) for CAA, 3.17 × 10^−4^ mm^2^s^−1^ (2.80–3.53) for AD and 2.72 × 10^−4^ mm^2^s^−1^ (2.45–2.98) for HCs; *post-hoc p* < 0.001 for CAA vs. AD, and CAA vs. HCs. The mean cortical thickness was significantly lower in the AD [2.27 mm (2.22–2.31)] vs. CAA [2.37 mm (2.33–2.40); *post-hoc p* < 0.001] and HCs [2.41 mm (2.37–2.44); *post-hoc p* < 0.001].

**Table 3:** Summary of quantitative MRI markers expressed as age-, sex-, and study site- adjusted least square means (95% confidence intervals).

MRI quantitative analysis (LS means)	CAA (*n* = 78)	AD (*n* = 33)	Controls (*n* = 70)	*p*-value
WMH volume, cm^3^	28.1 (23.3, 32.9)	10.5 (4.4, 16.7)	7.4 (3.0, 11.8)	<0.001 ^a,b^
WMH (% of eTIV)	1.85 (1.54, 2.16)	0.70 (0.30, 1.09)	0.48 (0.20, 0.76)	<0.001 ^a,b^
PSMD, 10^−4^ mm^2^s^−1^	4.02 (3.74, 4.30)	3.17 (2.80, 3.53)	2.72 (2.45, 2.98)	<0.001 ^a,b^
Mean cortical thickness, mm	2.37 (2.33, 2.40)	2.27 (2.22, 2.31)	2.41 (2.37, 2.44)	<0.001 ^a,c^
Estimated total intracranial volume (eTIV), cm^3^	1,507 (1,463, 1,549)	1,488 (1,433, 1,543)	1,478 (1,439, 1,518)	0.558
Cerebral cortex (% of eTIV)	29.4 (28.6, 30.1)	26.8 (25.9, 27.7)	30.4 (29.8, 31.1)	<0.001 ^a,b,c^
Cerebral white matter (% of eTIV)	29.5 (28.4, 30.7)	29.1 (27.6, 30.6)	29.9 (28.9, 30.9)	0.625
Thalamus (% of eTIV)	0.434 (0.420, 0.447)	0.422 (0.405, 0.439)	0.439 (0.426, 0.451)	0.234
Putamen (% of eTIV)	0.262 (0.250, 0.275)	0.265 (0.249, 0.281)	0.286 (0.275, 0.298)	0.004 ^b^
Caudate (% of eTIV)	0.218 (0.208, 0.229)	0.220 (0.207, 0.233)	0.223 (0.213, 0.232)	0.785
Globus pallidus (% of eTIV)	0.128 (0.122, 0.133)	0.128 (0.121, 0.135)	0.124 (0.119, 0.129)	0.499
Cerebellar cortex (% of eTIV)	3.49 (3.38, 3.60)	3.52 (3.38, 3.67)	3.54 (3.44, 3.64)	0.762
Cerebellar white matter (% of eTIV)	0.90 (0.86, 0.94)	0.88 (0.83, 0.93)	0.89 (0.86, 0.93)	0.843

All continuous data were presented as least-square means and 95% confidence intervals after adjusting for age, sex and study site. Reported *p* values were based on ANCOVA with Tukey’s test for *post-hoc* comparisons. *Post-hoc p* < 0.05 in ^a^ CAA vs AD, ^b^ CAA vs controls, and ^c^ AD vs controls.

AD, Alzheimer’s disease; CAA, cerebral amyloid angiopathy; eTIV, estimated total intracranial volume; LS, least-square; PSMD, peak width of skeletonized mean diffusivity; WMH, white matter hyperintensity.

**Figure 2:**
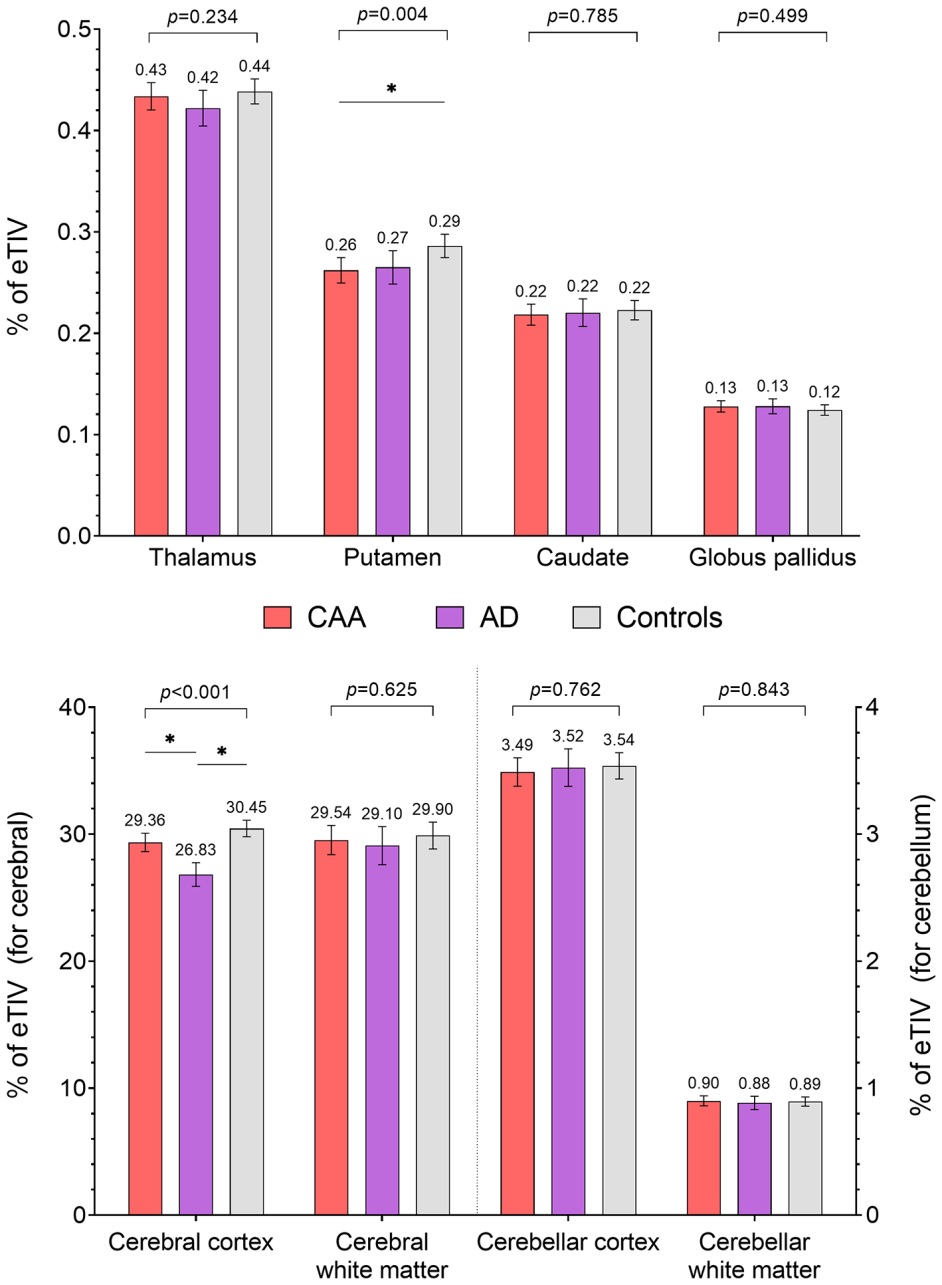
Comparisons of subcortical volumes (% of estimated total intracranial volume) between CAA, AD, and healthy controls (HC). * *Post-hoc p* < 0.05.

Overall, the segmented brain volume was negatively correlated with age (Pearson correlation = −0.269, *p* = 0.0003). Total intracranial volumes were comparable between the three groups; however, the cerebral cortical volumes differed between the three groups (Figure 2), being largest in the controls [30.4% of eTIV (29.8–31.1%)], followed by CAA [29.4% (28.6–30.1%)] then AD [26.8% (25.9–27.7%); overall *p* < 0.001, *post-hoc p* < 0.05 for all pairwise comparisons]. Cerebral white matter volumes were also comparable between the three groups [29.5% of eTIV (28.4–30.7%) vs. 29.1% (27.6–30.6%) vs. 29.9% (28.9–30.9%) for CAA, AD, and HCs, respectively; overall *p* = 0.63]. For deep nuclei structures (Figure 2), the putamen volume was smaller in CAA participants [0.262% of eTIV (0.250–0.275)] compared to HCs [0.286% (0.275–0.298); mean difference = −0.024% (−0.041 to −0.006%), *p* = 0.005]. This represented a mean difference of 0.36 ml or 8.4% of mean putamen volume. In contrast, CAA putamen volume was similar to AD participants [0.265% (0.249–0.281%); mean difference = −0.003% (−0.024 to 0.018%), *p* = 0.94]. Further adjusting for hypertension [mean difference between CAA and HCs = −0.031% (−0.050 to −0.013%), *p* = 0.0003] or WMH volume [mean difference between CAA and HCs = −0.023% (−0.043 to −0.003%), *p* = 0.03] as covariates did not change the results. Other deep nuclei such as the thalamus [CAA: 0.434% of eTIV (0.420–0.447%) vs. AD: 0.422% (0.405–0.439%) vs. HCs: 0.439% (0.426–0.451%)], caudate [CAA: 0.218% eTIV (0.208–0.229%) vs. AD: 0.220% (0.207–0.233%) vs. HCs: 0.223% (0.213–0.232%)], or globus pallidus [CAA: 0.128% of eTIV (0.122–0.133%) vs. AD: 0.128% (0.121–0.135%) vs. HCs: 0.124% (0.119–0.129%)] were not significantly different between the three groups (Figure 2).

Finally, there were no difference in volumes of the cerebellar cortex [3.49% of eTIV (3.38–3.60%) vs. 3.52% (3.38–3.67%) vs. 3.54% (3.44–3.64%) for CAA, AD, and HCs, respectively] or cerebellar white matter [0.90% of eTIV (0.86–0.94%) vs. 0.88% (0.83–0.93%) vs. 0.89% (0.86–0.93%) for CAA, AD, and HCs, respectively] between the three groups (Figure 2).

### Sensitivity analysis

To exclude the possibility of study site effect, we performed a sensitivity analysis of the quantitative MRI markers using only data from the Calgary site (*n* = 156). The relationships between the three groups were largely unchanged (Table 4). Importantly, the putamen volume remained the only subcortical structure that was different between CAA and HCs. To investigate whether the results differed between the left and right hemisphere, we compared the least square means on each hemisphere separately. The results still showed that the putamen was the only subcortical structure that differed between the groups (Supplementary Table 2).

**Table 4:** Summary of quantitative MRI markers (least square means) using only the Calgary data.

MRI quantitative analysis (LS means)	CAA (*n* = 72)	AD (*n* = 29)	Controls (*n* = 55)	*p*-value
WMH volume, cm^3^	26.2 (22.2, 30.1)	8.5 (2.5, 14.6)	5.9 (1.4, 10.5)	<0.001 ^a,b^
WMH (% of eTIV)	1.74 (1.49, 2.00)	0.56 (0.17, 0.95)	0.39 (0.10, 0.68)	<0.001 ^a,b^
PSMD, 10^−4^ mm^2^s^−1^	4.45 (4.22, 4.69)	3.63 (3.27, 4.00)	3.13 (2.85, 3.41)	<0.001 ^a,b^
Mean cortical thickness, mm	2.36 (2.33, 2.39)	2.27 (2.23, 2.30)	2.38 (2.36, 2.41)	<0.001 ^a,c^
Estimated total intracranial volume (eTIV)	1,497 (1,461, 1,532)	1,496 (1,442, 1,550)	1,473 (1,433, 1,513)	0.656
Cerebral cortex (% of eTIV)	30.1 (29.4, 30.7)	27.3 (26.4, 28.2)	30.9 (30.2, 31.6)	<0.001 ^a,c^
Cerebral white matter (% of eTIV)	28.6 (27.7, 29.5)	27.8 (26.4, 29.2)	29.3 (28.3, 30.3)	0.200
Thalamus (% of eTIV)	0.422 (0.411, 0.433)	0.407 (0.391, 0.424)	0.426 (0.413, 0.438)	0.234
Putamen (% of eTIV)	0.284 (0.274, 0.295)	0.286 (0.269, 0.302)	0.306 (0.294, 0.318)	0.028 ^b^
Caudate (% of eTIV)	0.232 (0.223, 0.240)	0.232 (0.219, 0.245)	0.234 (0.224, 0.244)	0.942
Globus pallidus (% of eTIV)	0.130 (0.126, 0.136)	0.130 (0.123, 0.137)	0.128 (0.123, 0.133)	0.690
Cerebellar cortex (% of eTIV)	3.58 (3.48, 3.67)	3.56 (3.42, 3.71)	3.59 (3.49, 3.70)	0.944
Cerebellar white matter (% of eTIV)	0.93 (0.90, 0.96)	0.90 (0.85, 0.95)	0.92 (0.89, 0.96)	0.613

All continuous data were presented as least-square means and 95% confidence intervals after adjusting for age, sex and study site. Reported *p* values were based on ANCOVA with Tukey’s test for *post-hoc* comparisons. *Post-hoc p* < 0.05 in ^a^ CAA vs AD, ^b^ CAA vs controls, and ^c^ AD vs controls.

AD, Alzheimer’s disease; CAA, cerebral amyloid angiopathy; eTIV, estimated total intracranial volume; LS, least-square; PSMD, peak width of skeletonized mean diffusivity; WMH, white matter hyperintensity.

### Associations with cognition

Furthermore, we examined the effects of these subcortical volumes on the cognitive function performance. No significant associations were observed between subcortical structures and cognitive function after adjusting for covariates (Table 5). The same findings were observed when analyzing each hemisphere separately (Supplementary Table 3).

**Table 5:** Associations between subcortical volumes and cognitive domain z score in all participants (*n* = 181).

Subcortical volume	Memory z score	Executive function z score	Processing speed z score
Thalamus	0.20 (−0.13, 0.52)	0.10 (−0.24, 0.44)	0.009 (−0.32, 0.34)
Putamen	−0.08 (−0.42, 0.27)	0.06 (−0.31, 0.42)	0.16 (−0.19, 0.51)
Caudate	0.07 (−0.35, 0.49)	0.10 (−0.35, 0.55)	−0.17 (−0.60, 0.25)
Globus pallidus	−0.40 (−1.18, 0.38)	−0.007 (−0.83, 0.82)	0.19 (−0.60, 0.98)
Cerebellar cortex	0.001 (−0.04, 0.04)	0.003 (−0.04, 0.04)	−0.003 (−0.04, 0.04)
Cerebellar white matter	0.01 (−0.10, 0.13)	−0.01 (−0.10, 0.13)	−0.03 (−0.14, 0.08)

The effect estimates represented the β and 95% CI from the multivariable linear regression, adjusting for age, female sex, education years, diagnostic group, and study site. The beta coefficients indicate the change in cognitive score for each volume increase of 0.1% of estimated total intracranial volume.

## Discussion

This cross-sectional analysis found that subcortical volumes in patients with CAA were not different from that of patients with AD or HCs, except for a smaller putamen volume in CAA compared with HCs. These results contrast with previous findings showing significant subcortical atrophy in CAA compared to HC participants and AD patients, including white matter (mean difference −2.38% of eTIV for CAA vs. controls and −1.57% for CAA vs. AD), basal ganglia (mean difference −0.13% and −0.05%), and cerebellum (Fotiadis et al., 2020, 2021; Horn et al., 2022).

A major difference between our result and those of previous studies is that we enrolled participants from the same cohort using a harmonized imaging protocol, while previous studies recruited CAA from a single hospital center and sampled AD and HCs from the ADNI database. Even though the AD and HCs were sampled from ADNI participants who were scanned using instruments made by the same manufacturer, there is nonetheless the possibility that bias may have been introduced. In our study, all participants were scanned on the same MRI system at each site. Furthermore, the results were similar when using data only from Calgary site. Apart from the study design, the demographic profile in CAA also varied. In our CAA participants, the mean age was 74 years and 35% were female; while in the previous cohort the mean age was 70 years and only 23% were female (Fotiadis et al., 2020, 2021; Horn et al., 2022). Additionally, our study used the updated Boston criteria v2.0 for diagnosis of CAA, while previous studies used modified Boston criteria v1.5. Therefore, the observed differences between studies may also reflect heterogeneity in the source populations.

The neuroimaging hallmarks of CAA are the presence of advanced cerebral SVD, especially haemorrhagic markers (Charidimou et al., 2016). Our results confirmed an overall higher burden of WMH, CMBs and cSS in CAA compared to AD patients and HCs. CAA participants also had impaired white matter integrity, reflected by higher PSMD compared to AD and HC participants (McCreary et al., 2020). In addition, the cerebral cortex volume and cortical thickness were lower in CAA than HCs, although not statistically significant. These findings and previous studies suggested that CAA exerts its effects on neurodegeneration by accumulating haemorrhagic and ischemic lesions, disrupting white matter connection and causing cerebral cortical atrophy (Smith, 2018); however, whether CAA affects subcortical structures remained elusive. In the current study, we did not observe a substantial reduction of subcortical volumes in CAA, although we cannot rule out the possibility of microstructural damage in these structures.

The putamen was the only subcortical structure that showed differences between CAA participants and HCs. One previous study found the basal ganglia volume including putamen, caudate, and globus pallidus were all smaller in the CAA group compared to AD and HC participants (Fotiadis et al., 2021). Although CAA pathology primarily affects the cortex, it may exert distant effects on subcortical brain regions through two possible mechanisms. First, chronic cortical vascular dysfunction and hypoperfusion of the distal internal borderzone may result in subcortical ischemia. Second, cortical degeneration may impair the afferent and efferent connections between cortex and subcortical structures; in turn leading to subcortical neurodegeneration (Fotiadis et al., 2021). However, these hypotheses do not explain why we found that only the putamen was affected in CAA, while other deep nuclei were spared. One possibility is that the striatum receives its blood supply from proximal deep perforating arterioles, which are vulnerable to the effects of hypertension, rather than the distal penetrating branches of cortical arteries (Fischl et al., 2004). Therefore, we can not rule out the possibility of superimposed hypertensive arteriosclerosis and lipohyalinosis affecting the basal ganglia, particularly as history of hypertension was more common in CAA. However, our results were unchanged after controlling for hypertension. Whether CAA is independently associated with putaminal atrophy requires more in-depth investigation.

Additionally, we did not find differences in white matter volume between three groups. One previous study demonstrated white matter atrophy in CAA and hypothesized that it may be caused by vascular dysfunction or CAA-related small lesions such as microbleeds or microinfarcts (Fotiadis et al., 2020). However, another study found superficial CMBs or CAA pathology in an elderly population to be associated with larger morphometric brain measurements, especially in white matter volume (Beaman et al., 2022). These contradictory finding could be due to impaired glymphatic drainage resulting from increased vascular Aβ in early stage CAA which may result in a relative increase in interstitial fluid, and in turn manifest as apparent increased white matter volume (Weller et al., 2008). Fundamentally, the heterogeneous results between studies may merely reflect the different symptom profiles and severity of recruited CAA participants.

Furthermore, we did not found associations between the subcortical structures and cognitive function. In fact, our previous study demonstrated that in CAA participants, the decline in cognitive performance was primarily mediated by PSMD, cortical thickness, and cerebrovascular reactivity to carbon dioxide (Durrani et al., 2023). Therefore, white matter integrity and cortical atrophy, rather than changes in the volume of subcortical structures, appear to be the main drivers of cognitive decline in CAA.

The methodologic strengths of this study include a standardized advanced neuroimaging protocol and analyses in an active cohort that simultaneously enrolled participants with CAA, AD, and healthy controls. Additionally, the sample size of CAA was relatively large compared to previous studies. There were several limitations of this study. First, the CAA participants were older than the other groups and aging itself is associated with brain atrophy (Peters, 2006). The healthy control group had more female participants. However, to account for these imbalances we reported least square means that were adjusted for age and sex. Furthermore, we did not find differences in subcortical volumes in either the unadjusted or the adjusted data (except for the putamen). Second, participants were enrolled from two study sites which using two different MRI systems, thus creating variation in the neuroimaging acquisition. However, the protocol was harmonized between sites and data were centrally processed. We also controlled for study site in the models. Importantly, results were unchanged when analyzing data from only Calgary, the major study site. Third, CAA participants had higher proportion of hypertension than the AD and controls, and hypertensive microangiopathy is known to affect subcortical structures including atrophy or morphometric changes (Strassburger et al., 1997; Jenkins et al., 2020). Nevertheless, we did not observe substantial impact of hypertension on the subcortical volumes in CAA after adjusting for hypertension in the model. Fourth, CAA and AD pathology may overlap especially in the aging population. We did not have information on amyloid and tau markers in our participants, therefore we could not apply the A/T/N classification system to diagnose AD (Jack et al., 2016). However, amyloid PET would be hard to interpret because it is abnormal in both CAA and AD (Farid et al., 2017). Nevertheless, we tried to reduce the possibility of overlap by: (1) recruiting CAA participants based on the relevant clinical symptoms and diagnosing them based on the Boston criteria v2.0 of probable CAA; and (2) excluding all participants in the AD group which had radiological features of probable CAA. Finally, although our sample size was larger than previous studies, it was still modest and did not allow for further subgroup analysis.

In conclusion, our study did not find substantial atrophy of subcortical structures in CAA compared to AD or healthy controls, except for a small difference in the volume of the putamen. Therefore, our findings suggest that CAA has a relatively mild effect on the subcortical nuclei. The differences between ours and previous studies may reflect heterogeneity in CAA presenting syndromes or severity. Further studies with multisite collaboration and larger sample sizes are necessary to help clarify this issue.

## Data availability statement

The datasets presented in this article are not readily available because De-identified study data will be shared upon reasonable request to the corresponding author. Requests to access the datasets should be directed to ES, eesmith@ucalgary.ca.

## Ethics statement

The studies involving human participants were reviewed and approved by the University of Calgary and University of Alberta Research Ethics Board. The patients/participants provided their written informed consent to participate in this study.

## Author contributions

ES, ZI, RF, GP, and RC designed the study. AB, CM, MG, RF, and GP arranged the imaging study. FS and ES did the imaging review. MK, AB, and CM performed the imaging analysis. C-HC performed the statistical analysis and drafted the manuscript. All authors revised and confirmed the final manuscript.

## Funding

This study was supported by the Canadian Consortium on Neurodegeneration of Aging [CCNA; Canadian Institutes of Health Research (CIHR) and partners: CCNA 137794, www.ccna-ccnv.ca] and the CIHR (MOP-142175 and FDN-154317), Brain Canada (MIRI2015-3994), Canadian Stroke Network, Heart and Stroke Foundation of Alberta, and the Alzheimer Society of Canada.

## Conflict of interest

ZI has received honoraria/consulting fees from Otsuka/Lundbeck and Roche, not relevant to this work. ES has received consulting fees from Eli Lilly.

The remaining authors declare that the research was conducted in the absence of any commercial or financial relationships that could be construed as a potential conflict of interest.

## Publisher’s note

All claims expressed in this article are solely those of the authors and do not necessarily represent those of their affiliated organizations, or those of the publisher, the editors and the reviewers. Any product that may be evaluated in this article, or claim that may be made by its manufacturer, is not guaranteed or endorsed by the publisher.

## Footnotes

^1^ http://surfer.nmr.mgh.harvard.edu/

^2^ http://www.psmd-marker.com

^3^ http://www.fmrib.ox.ac.uk.fsl
