# Supplementary material for: Subcortical volumes in cerebral amyloid angiopathy compared with Alzheimer’s disease and controls
Source: Front Neurosci. 2023 Apr 17;17:1139196. doi: 10.3389/fnins.2023.1139196 (PMC10149850; doi:10.3389/fnins.2023.1139196)
Supplement: Supplementary file 1 [file Table_1.docx]

**Supplemental Table 1**. MRI acquisition parameters.

|  | **FAVR I (GE Signa)** | | | | **FAVR II: Calgary (GE Discovery MR750)** | | | | **FAVR II: Edmonton (Siemens)** | | | |
| --- | --- | --- | --- | --- | --- | --- | --- | --- | --- | --- | --- | --- |
|  | **3D T1** | **FLAIR** | **DWI** | **SWI** | **3D T1** | **FLAIR** | **DWI** | **SWI** | **3D T1** | **FLAIR** | **DWI** | **SWI** |
| TE (ms) | 3 | 140 | 88 | 20 | 3 | 140 | 88 | 3.7-35 | 3 | 120 | 65 | 3.8-37 |
| TR (ms) | 7 | 9000 | 11000 | 30 | 7 | 9000 | 11000 | 30 | 2300 | 9000 | 6900 | 45 |
| Flip angle (°) | 11 | 90 | 90 | 15 | 8 | 125 | 90 | 15 | 9 | 165 | 90 | 17 |
| Acquisition matrix | 256×256 | 256×256 | 128×128 | 512×256 | 256×256 | 256×256 | 128×128 | 256×256 | 256×256 | 256×256 | 128×128 | 256×190 |
| Reconstructed voxel size (mm^3^) | 1 × 1 × 1 | 0.9375 × 0.9375 × 3.5 | 0.8594 × 0.8594 × 3.5 | 0.4688 × 0.4688 × 1 | 1 × 1× 1 | 0.9375 × 0.9375 × 3.0 | 2 × 2 × 2 | 0.5 × 0.5 × 1 | 1 × 1 × 1 | 0.9375 × 0.9375 × 3.0 | 2 × 2 × 2 | 0.9375 × 0.9375 × 2.0 |
| FOV (mm) | 256 | 240 | 220 | 240 | 240 | 240 | 256 | 240 | 256 | 240 | 256 | 240 |
| Other Options | TI=650 ms | TI=2250ms | NEX=2;11 dir; *b*=850 s/mm^2^ | ZIP2; ZIP512 | TI=400ms | TI=2250ms | 30 dir; *b*=1000 s/mm^2^ | 8 echoes;  IES=3.7 ms; ZIP2; ZIP512 | TI=900 ms | TI=2500ms | 30 dir, *b*=1000 s/mm^2^ | 7 echoes; IES = 5.5 ms |

Abbreviation: TE, echo time; TR, repetition time; TI, inversion time; FOV, field of view; FLAIR, fluid attenuated inversion recovery; DWI, diffusion weighted imaging; SWI, susceptibility weighed imaging; IES, interecho spacing.

**Supplemental Table 2. Estimated MRI subcortical volumes expressed as age-, sex-, and study site- adjusted least square means (95% confidence intervals), stratified by left and right side**

| **Structure** | **Side** | **CAA (n=78)** | **AD (n=33)** | **Controls (n=70)** | ***p* value** |
| --- | --- | --- | --- | --- | --- |
| Thalamus (% of eTIV) | L | 0.439 (0.423, 0.454) | 0.433 (0.413, 0.453) | 0.447 (0.443, 0.462) | 0.415 |
|  | R | 0.429 (0.416, 0.443) | 0.411 (0.393, 0.428) | 0.429 (0.417, 0.442) | 0.107 |
| Putamen (% of eTIV) | L | 0.261 (0.248, 0.274) | 0.268 (0.251, 0.285) | 0.286 (0.274, 0.298) | 0.007 ^b^ |
|  | R | 0.263 (0.250, 0.276) | 0.262 (0.246, 0.279) | 0.286 (0.274, 0.298) | 0.006 ^b,c^ |
| Caudate (% of eTIV) | L | 0.212 (0.200, 0.223) | 0.217 (0.203, 0.232) | 0.217 (0.207, 0.228) | 0.702 |
|  | R | 0.225 (0.214, 0.235) | 0.224 (0.210, 0.237) | 0.228 (0.219, 0.238) | 0.798 |
| Globus pallidus (% of eTIV) | L | 0.127 (0.121, 0.133) | 0.129 (0.121, 0.137) | 0.125 (0.120, 0.131) | 0.705 |
|  | R | 0.129 (0.123, 0.134) | 0.127 (0.120, 0.134) | 0.123 (0.118, 0.128) | 0.212 |
| Cerebellar cortex (% of eTIV) | L | 3.47 (3.36, 3.58) | 3.50 (3.36, 3.64) | 3.51 (3.41, 3.61) | 0.849 |
|  | R | 3.52 (3.40, 3.64) | 3.55 (3.40, 3.70) | 3.56 (3.46, 3.67) | 0.826 |
| Cerebellar white matter (% of eTIV) | L | 0.93 (0.89, 0.97) | 0.89 (0.84, 0.94) | 0.91 (0.87, 0.94) | 0.420 |
|  | R | 0.88 (0.84, 0.92) | 0.88 (0.82, 0.93) | 0.88 (0.84, 0.92) | 0.993 |

All continuous data were presented as least-square means and 95% confidence intervals after adjusting for age, sex and study site. Reported *p* values were based on ANCOVA with Tukey’s test for post-hoc comparisons. Post-hoc *P* < 0.05 in ^a^ CAA vs AD, ^b^ CAA vs controls, and ^c^ AD vs controls.

Abbreviations: AD, Alzheimer’s disease; CAA, cerebral amyloid angiopathy; eTIV, estimated total intracranial volume; LS, least-square

**Supplemental Table 3.** **Associations between subcortical volumes and cognitive domain z score**

| **Subcortical volume** | **Side** | **Memory Z score** | **Executive function Z score** | **Processing speed Z score** |
| --- | --- | --- | --- | --- |
| Thalamus | L | 0.11 (-0.15, 0.39) | 0.11 (-0.19, 0.39) | -0.009 (-0.29, 0.27) |
|  | R | 0.14 (-0.19, 0.47) | 0.07 (-0.28, 0.42) | 0.002 (-0.34, 0.34) |
| Putamen | L | 0.01 (-0.31, 0.34) | 0.03 (-0.32, 0.39) | 0.15 (-0.19, 0.49) |
|  | R | -0.17 (-0.51, 0.17) | 0.09 (-0.27, 0.45) | 0.20 (-0.15, 0.54) |
| Caudate | L | 0.18 (-0.19, 0.55) | 0.12 (-0.29, 0.53) | -0.12 (-0.51, 0.27) |
|  | R | -0.06 (-0.47, 0.35) | 0.08 (-0.36, 0.53) | -0.14 (-0.57, 0.29) |
| Globus pallidus | L | -0.23 (-0.92, 0.46) | 0.11 (-0.64, 0.85) | 0.34 (-0.38, 1.06) |
|  | R | -0.49 (-1.27, 0.30) | 0.001 (-0.85, 0.85) | 0.11 (-0.69, 0.92) |
| Cerebellar cortex | L | -0.001 (-0.04, 0.04) | 0.006 (-0.04, 0.05) | -0.001 (-0.04, 0.04) |
|  | R | 0.002 (-0.03, 0.04) | 0.006 (-0.03, 0.04) | -0.003 (-0.04, 0.03) |
| Cerebellar white matter | L | -0.03 (-0.13, 0.08) | 0.04 (-0.08, 0.15) | -0.04 (-0.15, 0.07) |
|  | R | 0.03 (-0.07, 0.13) | 0.02 (-0.08, 0.13) | -0.01 (-0.12, 0.09) |

The effect estimates represented the *β* and 95% CI from the multivariable linear regression, adjusting for age, female sex, education years, diagnostic group, and study site.
